# Hematological Parameters at Baseline: A Novel Prognostic Factor for Cervical Cancer Patients Undergoing Concurrent Chemoradiotherapy in South India

**DOI:** 10.7759/cureus.69461

**Published:** 2024-09-15

**Authors:** Monal Garg, Priya Bhati, Gautham Balaji, Ajay Sasidharan, Sruthi Kalavagunta, Sheejamol VS, Debnarayan Dutta

**Affiliations:** 1 Gynecological Oncology, Amrita Institute of Medical Sciences, Kochi, IND; 2 Radiation Oncology, Amrita Institute of Medical Sciences, Kochi, IND; 3 Biostatistics, Amrita Institute of Medical Sciences, Kochi, IND

**Keywords:** concurrent chemoradiation therapy, haematological indices, locally advanced cervical cancer, long-term prognosis, survival outcomes

## Abstract

Introduction

In cervical cancer treatment, neutrophil-to-lymphocyte ratio (NLR), platelet-to-lymphocyte ratio (PLR), and albumin-globulin ratio (AGR) are being studied as potential prognostic markers for predicting the effectiveness of concurrent chemoradiotherapy (CCRT). This study aims to investigate the relationship between these biomarkers and survival outcomes in cervical cancer patients undergoing CCRT.

Materials and methods

This retrospective study was conducted at Amrita Institute of Medical Sciences between January 2016 and December 2019. It included patients at any stage who received definitive CCRT and were followed for at least two years post-treatment. Patients who had initial surgery and those lost to follow-up were excluded.

Results

The study included 123 patients with a median age of 68. Most patients had stage IIB (39%) and squamous cell carcinoma (76.4%). With a median follow-up of 56 months, the five-year overall survival (OS) was 66.8%, progression-free survival (PFS) was 94%, and recurrence-free survival (RFS) was 81.2%. AGR (p = 0.001), NLR (p = 0.0001), and PLR (p = 0.001) were found to be significantly associated with OS, NLR (p = 0.002) and AGR (p = 0.001) significantly affected RFS, while only PLR (p = 0.02) significantly affected PFS on univariate analysis. NLR significantly impacted OS (p = 0.003) and RFS (p = 0.03) on multivariate analysis.

Conclusion

The results of our study showed that increased NLR and elevated levels of albumin indicate a higher likelihood of mortality. Furthermore, a higher NLR was linked to an increased probability of recurrence in patients with cervical cancer who received primary treatment with CCRT. Therefore, the identification of predictive biomarkers could significantly improve the assessment of progression risk, aiding in the selection of the most suitable treatment and personalized therapy.

## Introduction

The incidence of cervical cancer has decreased in the past 10 years. However, it still stands as the second most prevalent cancer among women in India, with a 9% incidence rate, according to GLOBOCAN (Global Cancer Observatory) 2022 [1]. The typical course of action for locally advanced cervical cancer (stage ≥ IB3 according to the International Federation of Gynecology and Obstetrics 2018 staging) involves undergoing concomitant chemoradiotherapy (CCRT) first, followed by receiving image-guided adaptive brachytherapy (IGABT) [2]. While the FIGO (International Federation of Gynecology and Obstetrics) staging is generally reliable for predicting outcomes before surgery, the clinical stage can be inaccurate, particularly in advanced cases [3,4]. Various factors, including patient characteristics, tumor attributes, and treatment-related variables, such as HPV (human papillomavirus) status, FIGO stage, tumor size, lymph node involvement, and histology, have been recognized as predictors of outcomes following radiation therapy or concurrent chemoradiotherapy [3,5]. However, these factors are typically assessed post-biopsy or surgery in histopathology reports. It is essential to have a preoperative test that is noninvasive and easily accessible to accurately forecast the survival probability and prognosis for cervical cancer [5].

Cancer can change hematological parameters due to inflammatory and immunosuppressive factors [1,6-8]. While the exact processes are not entirely understood, there is evidence indicating that it affects the prognosis of various cancer types, such as cervical cancer [9]. Elevated release of proinflammatory cytokines leads to widespread inflammatory reactions and changes in blood-related elements such as serum albumin, serum globulin, hemoglobin (Hb), neutrophil counts, lymphocyte counts, and platelet counts [10-15]. Recent studies suggest that certain pretreatment blood cell levels can indicate outcomes for cervical cancer patients. For example, higher neutrophil and monocyte levels are linked to poorer outcomes, while higher hemoglobin and lymphocyte levels are associated with improved outcomes. Ratios like the neutrophil-to-lymphocyte ratio (NLR), platelet-to-lymphocyte ratio (PLR), and albumin-globulin ratio (AGR) may help predict a patient's response to CCRT [14,15]. However, the results regarding the relation of these parameters with prognosis are inconsistent. Investigating readily available baseline tests for forecasting overall survival (OS) and progression-free survival (PFS) in resource-constrained countries such as India is essential. Therefore, we conducted this study to investigate the clinical predictive significance of pretreatment hematological factors and their association with clinical outcomes in cervical cancer.

## Materials and methods

Target population

In this study, 123 patients diagnosed with cervical cancer between January 2016 and December 2019 were included. The data was gathered retrospectively from the hospital's electronic database with the approval of the Institutional Ethics Committee (IEC-AIIMS-2024-GYNECONCO-179). The inclusion criteria were as follows: age above 18 years, histopathologically confirmed cervical cancer of FIGO 2018 staging IB or above [4], complete clinicopathological information, received definitive concurrent chemoradiotherapy, and having at least two years of follow-up either in person or via phone. Exclusion criteria included receiving a blood transfusion within two months before treatment; having a history of acute infection in the last three months; having chronic inflammatory conditions such as HIV infection, systemic lupus erythematosus (SLE), inflammatory bowel disease (IBD), rheumatoid arthritis (RA), or sepsis; and missing clinicopathological or laboratory information or being lost to follow-up.

Management protocol and follow-up

In our hospital, patients with locally advanced or advanced-stage cancer underwent routine blood tests. Imaging such as CE-MRI of the abdomen and pelvis, CECT of the whole abdomen and pelvis, or PET-CT scans were performed to determine the disease stage as per availability and convenience. Cervical biopsies were taken during pelvic examinations or guided by imaging, as appropriate. After confirming the stage and histopathology, patients received CCRT based on the institutional protocol. Routine blood tests were conducted one week before the treatment and four weeks after completion. Follow-ups occurred every three months for the first two years, every six months for the following three years, and annually after that. Patients underwent history-taking and pelvic examinations during each follow-up, and imaging was performed accordingly as indicated.

CCRT protocol

All patients received cisplatin-based chemotherapy. They began with at least one cycle of weekly intravenous cisplatin (40 mg/m^2^), aiming to complete five or six cycles during external beam radiotherapy (EBRT). If patients had impaired kidney function, they were given carboplatin instead of cisplatin. Treatment involved three-dimensional conformal radiation therapy (3DCRT) or image-guided radiation therapy (IGRT), with 1.8-2 Gy fractions administered up to five times per week. EBRT for the paraaortic region was planned based on imaging. High-dose-rate (HDR) brachytherapy (BT) was administered using an intracavitary technique. CT scans were used to plan both EBRT and BT. The total radiation therapy time was calculated from the first day of EBRT to the last day of BT. Starting from April 2019, all patients received treatment using IGRT.

Data collection

The following information was collected for each patient: basic demographic profile, age, comorbidities, ECOG (Eastern Cooperative Oncology Group) performance status, clinical presentation, BMI (body mass index), clinical stage, blood investigation parameters, histopathology, treatment given, chemotherapy drug used, and treatment response. Follow-up was conducted via phone until August 10, 2023. During follow-ups, the current status of the patients, progression, recurrences, progression/recurrence sites, and treatment received were noted. Hematological parameters such as Hb (hemoglobin) levels, WBC (white blood cell) count, neutrophil count, lymphocyte count, platelet count, serum albumin, and serum globulin levels were noted for all patients before the front-line treatment and four weeks after treatment completion. Additionally, the AGR, NLR, and PLR were calculated.

Statistical analysis

The data was analyzed using SPSS Statistics 20.0 software (IBM Corp., Armonk, NY). Categorical variables were described using frequencies and percentages, and ratios were compared using the Chi-square test. Survival curves were plotted using the Kaplan-Meier method, and Spearman’s rank correlation coefficient was utilized to test the correlation between different factors. OS, PFS, and RFS (recurrence-free survival) were calculated from the treatment onset date. Hematological parameters were associated with OS, PFS, and RFS. A p-value of <0.05 was considered statistically significant.

## Results

Basic demographic, clinical, and pathological characteristics

We enrolled 123 patients in our study who met our inclusion and exclusion criteria. Table 1 summarizes the initial patient characteristics and clinicopathological aspects of the disease.

**Table 1:** Demographic and clinical-pathological characteristics of patients included in the study (N = 123) The data has been represented as N and %. ECOG: Eastern Cooperative Oncology Group; FIGO: The International Federation of Gynecology and Obstetrics; PET: Positron emission tomography; CT: Computed tomography; MRI: Magnetic resonance imaging.

Variables	Category	N	Percentage
Age (years)	≤50	18	14.6
	>50	105	85.4
ECOG performance status	0	19	15.4
	1	92	74.8
	2	12	9.8
Comorbidities	No	53	43.1
	Yes	70	56.9
Clinical presentation	Postmenopausal bleeding	71	57.7
	Abnormal uterine bleeding	22	17.8
	Discharge	13	10.5
	Abdominal symptoms	10	8.1
	Asymptomatic	4	3.2
	Postcoital bleeding	2	1.6
	Urinary symptoms	1	0.8
Histology type	Squamous cell carcinoma	94	76.4
	Adenocarcinoma	15	12.2
	Poorly differentiated	14	11.4
FIGO 2018 stage	IB2	9	7.3
	IIA	8	6.5
	IIB	48	39
	IIIB	19	15.4
	IIIC1	13	10.5
	IIIC2	9	7.3
	IVA	17	13.8
Preoperative imaging	MRI	101	82.1
	PET-CT	22	17.9
Number of chemotherapy cycles	1	3	0.8
	2	4	3.2
	3	8	6.5
	4	2	1.6
	5	17	13.8
	6	77	62.6
Treatment course	Completed	104	84.5
	Stopped	19	15.4

Their median age was 61 (38-78) years, and the most common symptom reported was postmenopausal bleeding (57.7%). Other symptoms included menstrual irregularities (n = 22, 17.8%), abnormal vaginal discharge (n = 13, 10.5%), abdominal complaints (n = 10, 8.1%), postcoital bleeding (n = 2, 1.6%), and urinary symptoms (n = 1, 0.8%). However, four patients (3.2%) were symptom-free and were only diagnosed incidentally through imaging. Nineteen (15.4%) patients demonstrated an ECOG score of 0, while 92 (74.8%) patients had a score of 1, and 12 (9.8%) patients had a score of 2. The majority of patients presented with FIGO (the International Federation of Gynecology and Obstetrics) stage IIB (n = 48, 39%), followed by stage IIIC (n = 21, 17.8%), stage IIIB (n = 19, 15.4%), stage IVA (n = 17, 13.8%), stage IB2 (n = 9, 7.3%), and stage IIA (n = 8, 6.5%). Squamous cell carcinoma, adenocarcinoma, and poorly differentiated carcinoma were observed in 94 (76.4%), 15 (12.2%), and 14 (11.4%) patients, respectively.

Treatment received

All patients who underwent CCRT were administered at least one cycle of cisplatin- or carboplatin-based chemotherapy. Cisplatin was given to most patients (n = 104, 84.6%), whereas carboplatin was given to 19 (15.3%) patients. The patients were given a median radiation dose of 46 Gy, ranging from 42 to 52.4 Gy. Most patients received either 45 Gy (n = 64, 52%) or 50.4 Gy (n = 59, 47.9%). The median duration of EBRT was 38 days, with a range of 25-64 days. The median total cumulative dose for the complete radiation treatment was 84 Gy, administered over 48 days, with a range of 28-102 days. The median period between biopsy-based diagnosis and the commencement of CCRT was 26 days, ranging from 12 to 42 days. The treatment course was completed by 84.5% (n = 104) of patients, with 15.4% (n = 19) of patients experiencing treatment withholdings due to intolerance. Additionally, 13 patients received blood transfusions. All patients received BT, with a median dose of 38 Gy.

Hematological parameters and their calculated cut-off values

The cut-off values for each parameter were determined using the ROC (receiver operating characteristic) curve, as depicted in Table 2.

**Table 2:** Hematological parameters with cut-off values analyzed using receiver operating curve analysis in patients with cervical cancer and treated with CCRT (N = 123) p-value is considered significant if <0.05. NLR: Neutrophil-to-lymphocyte ratio; PLR: Platelet-to-lymphocyte ratio; AGR: Albumin-globulin ratio; CCRT: Concurrent chemoradiotherapy.

Category	NLR	PLR	Albumin (g/dl)	Globulin (g/dl)	AGR
Area under curve	0.875	0.647	0.649	0.654	0.696
95% Confidence interval	0.804-0.946	0.542-0.752	0.548-0.749	0.548-0.760	0.596-0.795
Standard error	0.036	0.054	0.05	0.054	0.051
Sensitivity	91.4%	70.6%	80%	65.7%	51.4%
Specificity	84.4%	60.2%	47.7%	59.1%	78.4%
Cut-off value	4.28	150	4.28	3.32	1.08
P-value	0.0001	0.012	0.01	0.008	0.001

The cut-off value for Hb was 11.4 g/dl, WBC was 8.4 K/uL, albumin was 4.28 g/dl, globulin was 3.32 g/dl, NLR was 4.28, PLR was 150, and AGR was 1.08. Using these cut-off values, survival analyses were compared between the groups, as listed in Table 3.

**Table 3:** Univariate and multivariate analysis for correlation between clinical-pathological and hematological parameters with survival outcomes (N = 123) The data has been represented as N and %. *p-value considered significant (p < 0.05). **p-value considered highly significant (p < 0.001). (Ref)^: Reference value for calculating hazard ratio. OS: Overall survival; PFS: Progression-free survival; RFS: Recurrence-free survival; HR: Hazard ratio; CI: Confidence interval; UVA: Univariate analysis; MVA: Multivariate analysis; WBC: White blood cell; NLR: Neutrophil-to-lymphocyte ratio; PLR: Platelet-to-lymphocyte ratio; AGR: Albumin-globulin ratio.

Category (N = 123)	Subcategories	N	Percentage	OS			PFS			RFS		
				HR (95% CI)	UVA	MVA	HR (95% CI)	UVA	MVA	HR (95% CI)	UVA	MVA
Age (years)	≤50 (Ref)^	18	14.6	6.94 (0.94-10.45)	0.05*	0.03*	1.06 (0.12-8.81)	0.09	-	1.20 (0.24-3.91)	0.07	-
	>50	105	85.4									
Stage	≤IIB (Ref)^	65	52.8	2.30 (1.15-4.50)	0.01*	0.65	5.42 (0.17-10.82)	0.05*	0.32	3.35 (1.17-9.52)	0.16	-
	>IIB	58	47.1									
Hemoglobin (g/dL)	≤11.5	52	42.2	1.83 (0.94-3.56)	0.07	0.09	1.43 (0.11-4.32)	0.42	-	1.41 (0.54-3.66)	0.08	-
	>11.5 (Ref)^	71	57.7									
WBC (× 10^9^/L)	≤8.4 (Ref)^	75	60.9	1.16 (0.59-2.26)	0.60	-	2.05 (0.45-9.17)	0.34	-	1.80 (0.69-4.67)	0.06	-
	>8.4	48	39									
NLR	≤4.28 (Ref)^	77	62.6	21.4 (8.8-38.2)	<0.001**	0.003*	2.56 (0.57-11.4)	0.19	-	4.24 (1.6-11.2)	0.004*	0.014*
	>4.28	46	37.4									
PLR	≤150 (Ref)^	67	54.5	3.10 (1.51-6.34)	0.002*	0.32	7.56 (0.91-14.2)	0.02*	0.06	2.20 (0.83-5.70)	0.10	-
	>150	56	45.5									
Albumin (g/dL)	≤4.28	74	60.2	2.96 (1.29-6.80)	0.01*	0.04*	0.91 (0.2-4.08)	0.90	-	1.88 (0.66-5.35)	0.20	-
	>4.28 (Ref)^	49	39.8									
Globulin (g/dL)	≤3.32 (Ref)^	64	52.0	2.79 (1.37-5.66)	0.004*	0.37	10.4 (0.45-28.4)	0.005*	0.06	4.81 (1.56-14.83)	0.006*	0.018*
	>3.32	59	48.0									
AGR	≤1.13 (Ref)^	38	30.9	3.16 (1.61-6.20)	<0.001**	0.69	5.8 (0.78-12.31)	0.10	-	4.34 (1.64-11.47)	0.003*	0.36
	>1.13	85	69.1									

OS and its association with hematological parameters

**Figure 1:**
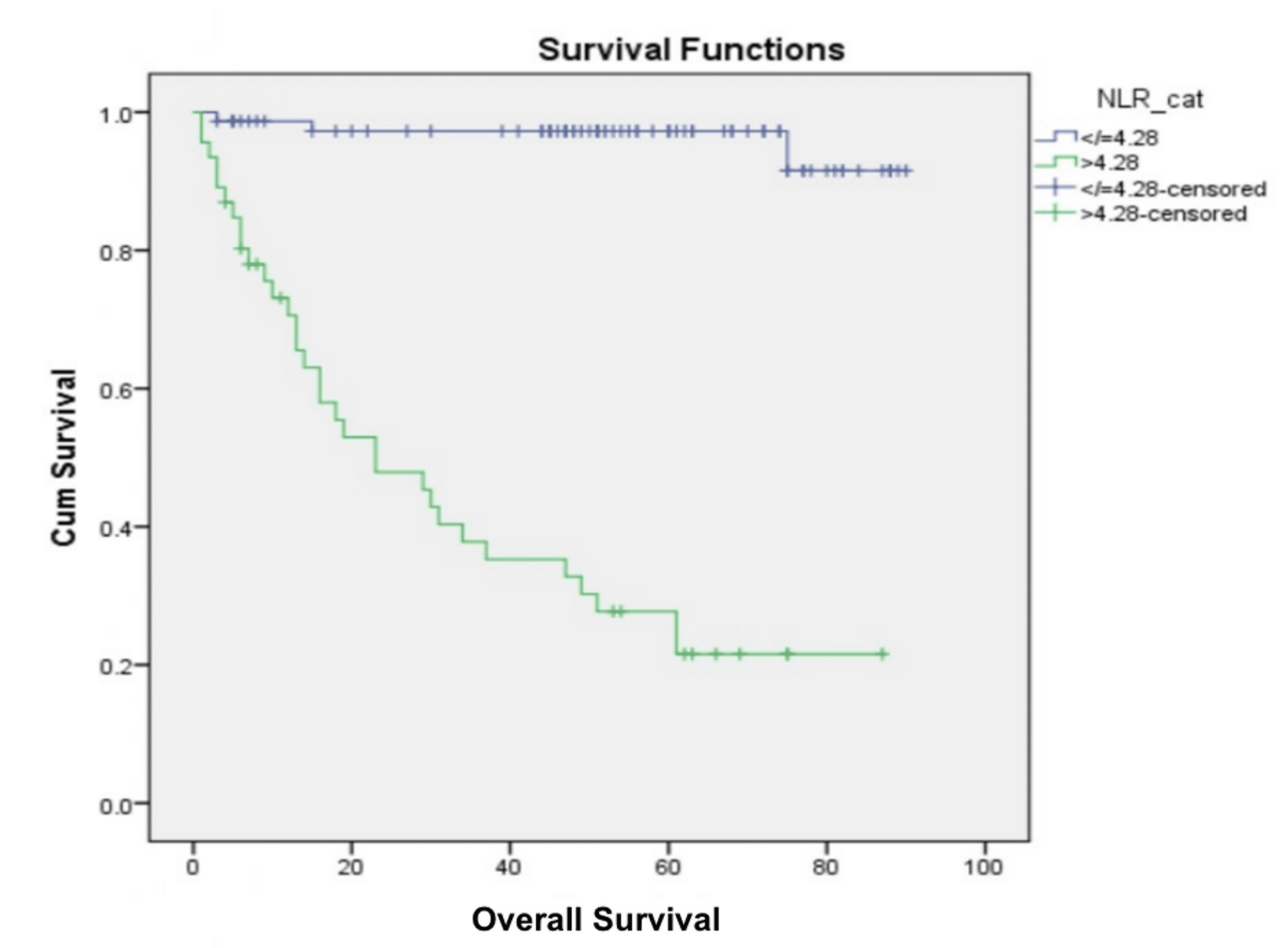
Kaplan-Meier curve showing overall survival outcomes using NLR cut-off value of 4.28 NLR: Neutrophil-to-lymphocyte ratio.

The median follow-up duration was 56 months (38-129 months). Of 123 patients, 35 (28.4%) expired within the defined follow-up period. The five-year OS rate was 66.8%. Univariate analysis explored the connection between various factors and OS. Patients aged ≤50 years (p = 0.05), with FIGO stage ≤ IIB (p = 0.01), serum albumin levels > 4.28 g/dL (p = 0.01), serum globulin levels ≤ 3.32 (p = 0.004), AGR > 1.13 (p = 0.001), NLR ≤ 4.28 (p = <0.001) (Figure 1), and PLR ≤ 150 (p = 0.002) exhibited significantly higher OS rates. No significant associations were found with WBC count (p = 0.60) or Hb levels (p = 0.07). In a Cox proportional hazards model, age (p = 0.03), NLR (p = 0.003), and serum albumin levels (p = 0.04) showed independent effects on OS.

PFS and its association with hematological parameters

**Figure 2:**
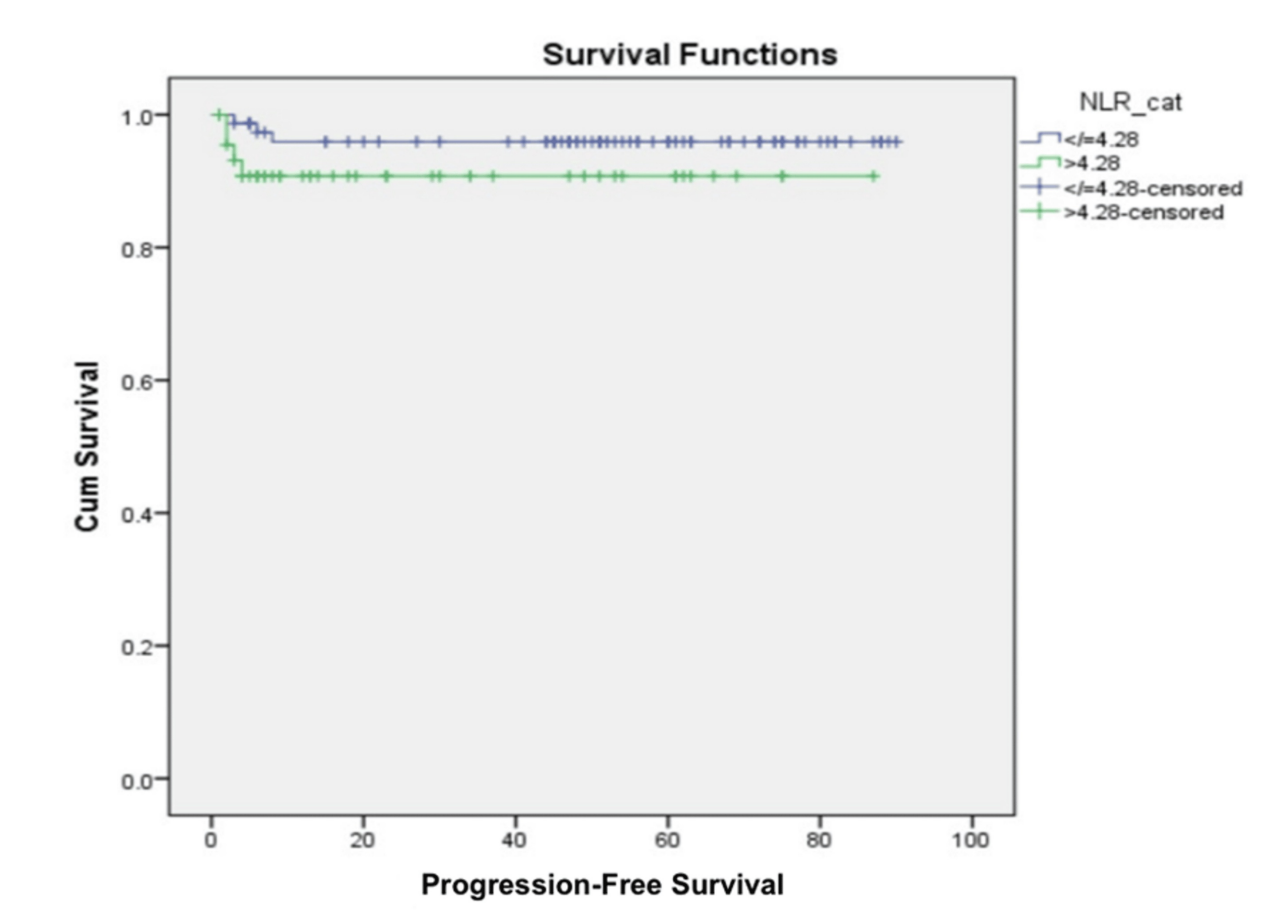
Kaplan-Meier curve showing progression-free survival outcomes using NLR cut-off value of 4.28 NLR: Neutrophil-to-lymphocyte ratio.

Eight patients (6.5%) experienced disease progression during the treatment, with six patients having stage IVA disease and two patients with stage IIIC disease. The five-year PFS rate stood at 94%. Upon analysis, only stage (p = 0.05) and PLR (p = 0.02) showed a significant association with PFS. In contrast, age (p = 0.09), Hb (p = 0.42), WBC count (p = 0.34), serum albumin (p = 0.90), serum globulin (p = 0.50), AGR (p = 0.10), and NLR (p = 0.19) displayed insignificant associations (Figure 2). Interestingly, on multivariate analysis, PLR (p = 0.06) was not identified as an independent risk factor affecting PFS.

RFS and its association with hematological parameters

**Figure 3:**
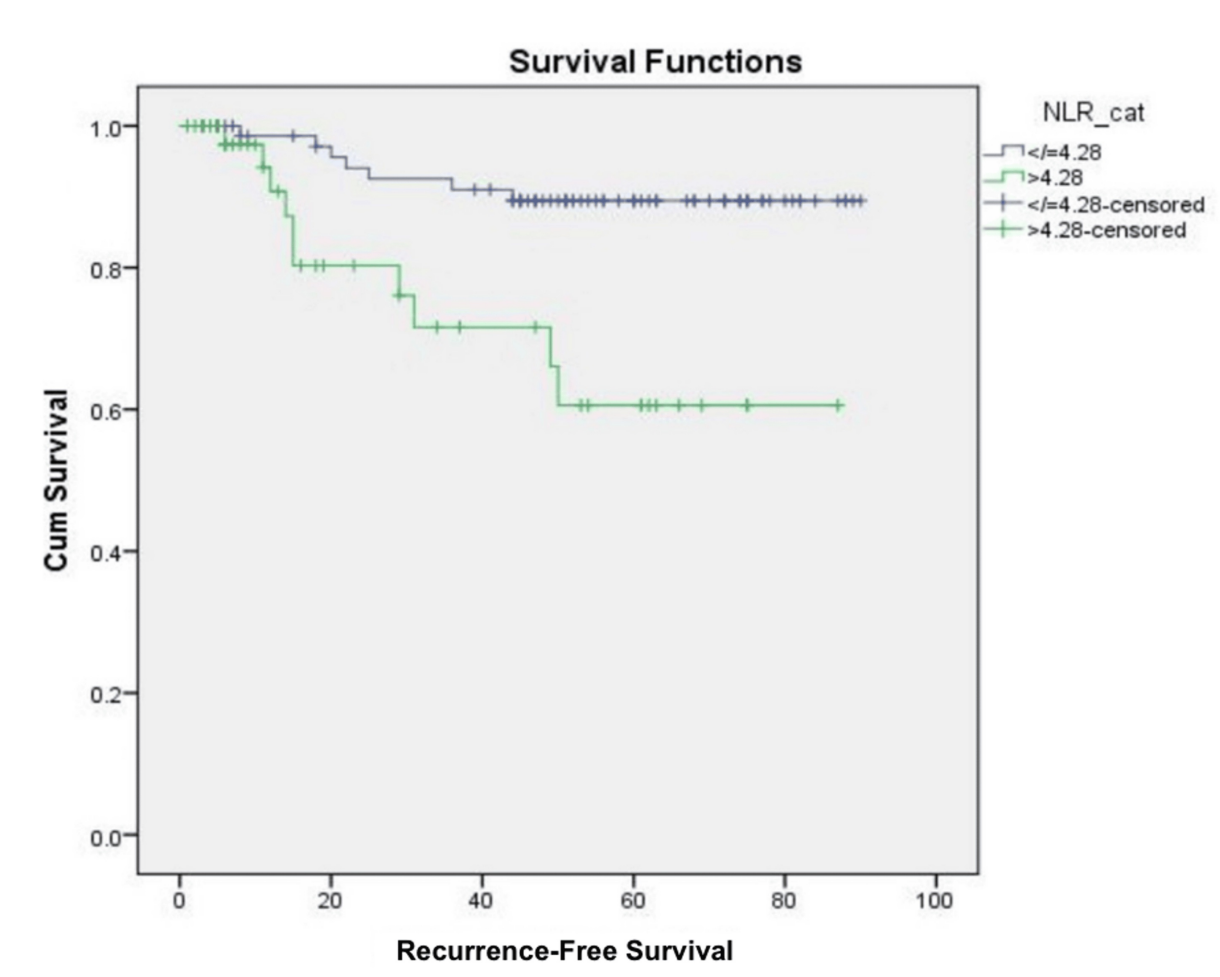
Kaplan-Meier curve showing recurrence-free survival outcome using NLR cut-off value of 4.28 NLR: Neutrophil-to-lymphocyte ratio.

After receiving treatment, 17 patients (13.8%) experienced recurrences during follow-up. The five-year RFS rate was 81.2%. Most recurrences occurred at distant sites (n = 13, 76.4%), while others were observed in loco-regional areas (n = 8, 47%) and the abdominal regions (n = 2, 11.7%). The recurrences were managed using various chemotherapy drugs, including the paclitaxel-carboplatin combination (n = 4, 23.5%); paclitaxel-carboplatin and bevacizumab combination (n = 2, 11.7%); cisplatin, ifosfamide, and paclitaxel (TIP) combination (n = 3, 17.6%); gemcitabine and carboplatin combination (n = 1, 5.8%); and whole-brain radiation therapy (WBRT) for brain metastasis (n = 3, 17.6%). It was observed that serum globulin (p = 0.006), AGR (p = 0.003), and NLR (p = 0.004) significantly impacted the univariate analysis (Figure 3). However, only serum globulin (p = 0.018) and NLR (p = 0.014) affected RFS independently in the multivariate analysis.

Subgroup analysis for radiation techniques and its influence over hematological parameters 

In the past, 3DCRT was the primary radiotherapy technique at our center. However, in 2019, we began using IGRT. The patients treated with radiotherapy through 3DCRT or IGRT accounted for 98 (79.6%) and 25 (20.3%) of the total, respectively. Notably, detailed pelvic volume information was available for only 25 IGRT patients. No variation was observed in hematological parameters with IGRT.

## Discussion

Systemic inflammation plays a crucial role in shaping the tumor environment by involving cytokines, inflammatory cells, and chemokines [16,17]. Notably, neutrophils and platelets aid in tumor expansion and migration. Lymphocytes assist in combating cancer cells [18]. Therefore, elevated NLR and PLR levels may indicate tumor hostility and reflect the defense mechanism of an individual. Several studies have been conducted to evaluate NLR and/or PLR as predictive factors in cervical cancer [19-34]. Table 4 summarizes previous studies assessing the effect of PLR or NLR on survival outcomes in cervical cancer patients.

**Table 4:** Review of literature comparing previous studies assessing the effect of PLR or NLR on survival outcomes in cervical cancer patients *p-value < 0.05 was considered significant. SCC: Squamous cell carcinoma; AC: Adenocarcinoma; CCRT: Concurrent chemoradiotherapy; BT: Brachytherapy; RH: Radical hysterectomy; PLND: Pelvic lymphadenectomy; NACT: Neoadjuvant chemotherapy; RT: Radiotherapy; F/B: Followed by; PLR: Neutrophil-to-lymphocyte ratio; NLR: Neutrophil-to-lymphocyte ratio; OS: Overall survival; DFS: Disease-free survival; PFS: Progression-free survival; N/A: Not available; NS: Non-significant; LRR: Lower relative risk.

Study	Type of the study	Study duration	Sample size (N)	Stages	Histology	Treatment	PLR cut-off	NLR cut-off	Median follow-up period (months)	Univariate analysis		Multivariate analysis	
										PLR	NLR	PLR	NLR
Wang et al. [19]	Retrospective, single-center	1999-2010	111	IB2-IIB	All	NACT F/B RH+PLND	142.2	2.4	N/A	NS	NS	NS	NS
Zhang et al. [20]	Retrospective, single-center	2005-2008	460	I-II	SCC, AC	RH+PLND	150.9	2.2	69	NS	OS, PFS	NS	OS
Jain et al. [13]	Retrospective	2005-2013	56	I-IV	SCC	RT/CCRT	N/A	2.5	NR	N/A	OS, PFS	N/A	OS, PFS
Chen et al. [21]	Retrospective, single-center	2006-2009	407	IB1–IIA	All	Any	143.47 (OS) 152.02 (RFS)	2.09 (OS) 2.59 (RFS)	NR	OS, RFS, LMN	OS, RFS	OS, RFS, LMN	OS, RFS
Onal et al. [22]	Retrospective, single-center	2006-2014	235	IB2-IVA	SCC, AC	CCRT	133.02	3.03	31.7	OS	OS, PFS	NS	OS, PFS
Zhu et al. [11]	Retrospective, single-center	2003-2008	96	IA–IV	AC	RT/CCRT	158,	2.32	N/A	OS (III-IV)	OS, PFS	NR	NR
Choi et al. [12]	Retrospective, single-center	2012-2014	339	I-IV	SCC	RH+PLND	138.8	2.5	44	OS, PFS	NS	PFS	NS
Nuchpramool et al. [23]	Retrospective, single-center	2001-2016	484	IA2-IB1	All	RH+PLND	NS	NS	56.9	NS	NS	NS	NS
Holub and Biete [24]	Retrospective, single-center	2009-2016	151	I-IV	All	Any	210	3.8	43.8	OS	OS	NS	NS
Huang et al. [25]	Meta-analysis	2010-2013	2804	I-IV	All	Any	N/A	NR	N/A	N/A	OS, PFS	N/A	OS, PFS
Wu et al. [26]	Meta-analysis	2012-2016	2452	I-IV	All	Any	N/A	N/A	N/A	N/A	OS, PFS	N/A	OS, PFS
Ittiamornlert and Ruengkhachorn [27]	Retrospective, single-center	2006-2017	355	IVB	All	NACT	N/A	3.6	NR	N/A	OS, PFS	N/A	N/A
Trinh et al. [28]	Retrospective, single-center	2008-2019	99	I-IV	All	Any	186.93	1.65	48.9	NS	OS, PFS	NS	OS
Jonska-Gmyrek et al. [10]	Retrospective, single-center	2008-2018	148	I-IV	All	CCRT+BT	148.89	2.34	75	Combined NLR and PLR significantly associated with OS and DFS		Combined NLR and PLR significantly associated with OS and DFS	
Du et al. [29]	Retrospective, single-center	2012-2017	203	I-IIA	SCC, AC, ASC	RH	N/A	3.75	NR	N/A	OS, PFS	N/A	NR
Sabyasachi et al. [30]	Retrospective, single-center	2017-2019	208	IB3-IIIC1	SCC	CCRT+BT	140.6	2.45	N/A	LRR	LRR	LRR	LRR
Zhao et al. [31]	Retrospective, single-center	2008-2018	202	I-IV	All	RT/CCRT	N/A	3.029	71	N/A	OS, PFS	N/A	OS, PFS
Gao et al. [32]	Retrospective, single-center	2001-2016	110	I–IV	All	RH+PLND	186.88	N/A	N/A	OS	N/A	OS	N/A
Jin et al. [33]	Retrospective, single-center	2012-2016	190	IB2-IVA	N/A	CCRT+BT	N/A	2.52	46	N/A	OS, PFS	N/A	OS, PFS
Kumar et al. [34]	Retrospective, single-center	2003-2017	1051	IB2-IVA	All	CCRT+BT	N/A	N/A	69	OS, DFS	N/A	OS, DFS	N/A

Despite varying inclusion criteria such as different cancer stages, histologies, treatments, and cut-off values, most of these studies separately analyzed NLR and PLR and consistently identified a correlation with survival outcomes. Our research also observed that a high NLR value (>4.28) was associated with lower OS and RFS in univariate and multivariate analyses. Similarly, a higher PLR (>150) was linked to a significant decrease in OS as well as the PFS.

The albumin concentration is a crucial clinical marker, providing insights into an individual's nutritional status. Low levels of albumin can negatively impact metabolism and the function of immune cells by reducing their effectiveness. In addition to its role in the immune system, albumin also regulates the inflammatory response by acting as an antioxidant agent in the development of tumors [35]. A decrease in serum albumin levels has been linked to an increased inflammatory response to cancer cells and the heightened release of various cytokines that lead to progress in tumor infiltration [36]. Few studies have shown an inverse relationship between serum albumin and oncological consequences [37]. However, Yoshino et al. did not find any connection between albumin levels and survival rates [38]. Our research, on the other hand, uncovered a significant impact of decreased albumin levels on OS in both univariate (p = 0.01) and multivariate analyses (p = 0.04).

Globulin is another marker for immune and inflammatory status, and higher levels have been linked to advanced cancer, leading to a negative impact on the immune system of cancer patients [38]. Yoshino et al. found that higher serum globulin levels were significantly linked to lower OS in patients with cervical cancer [38]. However, other studies did not find a significant correlation between elevated globulin levels and treatment outcomes in cervical cancer [37]. On the other hand, our study observed that higher globulin levels were associated with poorer OS, PFS, and RFS in univariate analyses and poorer RFS in multivariate analyses. This discrepancy could be due to a false increase in serum globulin levels, possibly caused by urinary tract infections or other inflammatory processes resulting from infections. Some researchers have calculated that the lower AGR denotes lower survival for cervical cancer patients [39]. However, other studies failed to demonstrate that AGR was an independent predictor for survival [38]. Our study, however, only showed a low AGR association with poor OS in univariate analysis.

Other significant poor prognostic factors associated with OS were age > 50 years and stage > IIB in our study. Age above 50 years was associated with a poorer OS in the multivariate analysis. However, pretreatment decreased hemoglobin levels, and higher WBC counts did not influence OS or PFS. A study conducted by Lee et al. found that, in their analysis, cancer stage, age, and pretreatment hemoglobin levels were linked to OS when looked at individually, but only the cancer stage was linked to poorer overall survival in their combined analysis [10].

Our study showed that IGRT administered to the patients did not affect hematological parameters. However, to our knowledge, no other research has demonstrated the effect of radiation dose on NLR, PLR, albumin, globulin, and AGR levels.

Strengths and limitations

Our study identified a strong association between elevated NLR levels and increased albumin levels with reduced OS and RFS, as confirmed by multivariate analysis. We also calculated AGR, a parameter rarely studied in previous literature. The study was conducted in a South Indian state with one of the lowest age-standardized cervical cancer incidence rates, at 9.35% per 100,000 women, compared to other states [40]. There is limited research in India on the prognosis of cervical cancer using hematological parameters, making our study an essential contribution to the development of noninvasive markers for highlighting the prognosis of locally advanced and advanced-stage cervical cancer patients.

However, our study has some limitations. First, the study was retrospective with a limited sample size. We also ruled out infection based solely on the patient's history without conducting prior tests, which could have potentially skewed the data on serum globulin levels. Additionally, we included all histologies and advanced stages, which could have influenced the survival outcomes. Lastly, our study was conducted in a single institution. Further studies involving more stratified, larger populations and multicenters are needed to reduce selection bias and confirm our findings.

## Conclusions

Our research revealed that higher NLR and elevated albumin levels predict a greater risk of mortality, and a higher NLR was associated with higher chances of recurrence in cervical cancer patients who underwent primary treatment with CCRT. Thus, identifying predictive biomarkers could significantly enhance the assessment of progression risk, assisting in the selection of the most appropriate treatment and personalized therapy. Further research is crucial to validate our findings and gain a deeper understanding of how they can be applied in clinical practice.
